# Hepatitis E Virus and Implications for Blood Supply Safety, Australia

**DOI:** 10.3201/eid2011.140412

**Published:** 2014-11

**Authors:** Ashish C. Shrestha, Clive R. Seed, Robert L.P. Flower, Kelly M. Rooks, Anthony J. Keller, Robert J. Harley, Hiu-Tat Chan, Jerry A. Holmberg, Helen M. Faddy

**Affiliations:** Australian Red Cross Blood Service, Kelvin Grove, Queensland, Australia (A.C. Shrestha, R.L.P. Flower, K.M. Rooks, R.J. Harley, H.M. Faddy);; The University of Queensland, St. Lucia, Queensland, Australia (A.C. Shrestha, H.M. Faddy);; Australian Red Cross Blood Service, Osborne Park, Western Australia, Australia (C.R. Seed, A.J. Keller);; Australian Red Cross Blood Service, West Melbourne, Victoria, Australia (H.-T. Chan);; Grifols, Emeryville, California, USA (J.A. Holmberg)

**Keywords:** Hepatitis E virus, seroprevalence, antibody, transmission, Australia, blood donor, risk factor, safety, blood supply, viruses

**To the Editor:** Hepatitis E virus (HEV) is an emerging public health concern for industrialized countries (*1*). Although HEV infection has been associated with travel to countries where the virus is endemic, cases of autochthonous HEV are increasing (*2*). Detection of HEV RNA in blood donations in the United Kingdom, Germany, the Netherlands, Japan, and China and accumulating reports of HEV transmitted through blood transfusion highlight the potential risk this virus poses to blood supply safety (*1*–*4*).

In Australia, where most HEV infections are associated with travel (*5*), an average of 25 HEV cases occurred each year during 1999–2013 (http://www9.health.gov.au/cda/source/rpt_3.cfm). HEV infection is nationally notifiable in Australia, but the presence of subclinical infections and the lack of recent seroprevalence studies have prevented the accurate estimation of HEV incidence and population exposure. Thus, we examined HEV seroprevalence in a cohort of Australian blood donors, assessed risk factors for exposure, and used the data to examine the effectiveness of current blood safety strategies for the management of HEV in Australia.

Plasma samples (n = 3,237) were collected from donors during August–September 2013. Information on age, sex, state of residence, new/repeat donor status, and overseas travel disclosure was obtained. Details of any relevant blood donation deferral (malaria, diarrhea) applied on previous donation attempts were also collected. Application of a specific malaria deferral code is routine for donors disclosing travel to a malaria-endemic country, and a diarrhea deferral applies when a donor reports having had diarrhea (of viral or unknown cause) 1 week before any attempted donation. 

All samples were tested for HEV IgG by using the Wantai HEV-IgG ELISA (Beijing Wantai Biologic Pharmacy Enterprise Co., Ltd, Beijing, China). Positive samples were tested for HEV IgM by using the Wantai HEV-IgM ELISA and for HEV RNA by using a prototype transcription-mediated amplification assay (Hologic Inc., San Diego, CA, USA).

Of 3,237 samples, 194 (5.99%) were positive for HEV IgG (95% CI 5.18–6.81). Compared with estimates from previous studies that used the Wantai ELISA (*6*–*9*), our estimate is comparable to those reported from Scotland (4.7%) and New Zealand (4.2%) but lower than those from the United States (18.8%) and southwestern France (52.5%). Considerable debate exists regarding the sensitivity and specificity of HEV detection methods (*2*,*10*); however, on the basis of studies in France and the United Kingdom (*9*,*10*), we believe that the measured seroprevalence in our study is accurate.

Our findings showed an increased seroprevalence of HEV associated with previous malaria deferral, diarrhea deferral, and age (multivariate logistic regression) (Table), the latter of which is consistent with previous findings (*9*). IgG seropositivity was also higher (7.73%) in donors who had traveled to a malaria-endemic country. HEV is often endemic to malaria-endemic countries (http://wwwnc.cdc.gov/travel/yellowbook/2014); however, the HEV exposure status of travelers is unknown before departure, so the exact place of exposure cannot be determined. Furthermore, 3.37% of donors in our study had evidence of previous HEV exposure; these donors had not reported travel outside Australia, so they may have acquired HEV locally. Because subclinical HEV infection is possible, persons infected locally may not be identified by the current donor screening questionnaire and thus pose a potential risk to blood supply safety.

**Table:** HEV (IgG) seroprevalence, and risk factors for exposure, in Australian blood donors*

Risk factor	No. tested	HEV IgG seropositive			Univariate analysis			Multivariate analysis	
		No.	% (95% CI)		OR (95% CI)	p value		OR (95% CI)	p value
Sex									
F	1,453	78	5.37 (4.21–6.53)		†	†		–	–
M	1,784	116	6.50 (5.36–7.65)		1.23 (0.91–1.65)	0.177		–	–
Age, y						0.000			0.000
<25	564	14	2.48 (1.20–3.77)		†	†		†	†
25–34	569	13	2.28 (1.06–3.51)		0.92 (0.43–1.98)	0.827		0.82 (0.38–1.77)	0.61
35–44	510	22	4.31 (2.55–6.08)		1.77 (0.89–3.5)	0.100		1.72 (0.87–3.42)	0.118
45–54	666	40	6.01 (4.20–7.81)		2.51 (1.35–4.66)	0.004		2.43 (1.30–4.52)	0.005
55–64	673	68	10.10 (7.83–12.38)		4.41 (2.46–7.94)	0.000		4.18 (2.32–7.54)	0.000
≥65	255	37	14.51 (10.19–18.83)		6.67 (3.54–12.58)	0.000		6.09 (3.21–11.55)	0.000
State of residence						0.580		–	–
ACT	406	25	6.16 (3.82–8.50)		†	†		–	–
NSW	405	23	5.68 (3.42–7.93)		0.92 (0.51–1.64)	0.773		–	–
NT	407	26	6.39 (4.01–8.76)		1.04 (0.59–1.83)	0.892		–	–
QLD	402	18	4.48 (2.46–6.50)		0.71 (0.38–1.33)	0.289		–	–
SA	404	32	7.92 (5.29–10.55)		1.31 (0.76–2.25)	0.328		–	–
TAS	401	20	4.99 (2.86–7.12)		0.80 (0.43–1.46)	0.800		–	–
VIC	411	23	5.60 (3.37–7.82)		0.90 (0.50–1.62)	0.733		–	–
WA	401	27	6.73 (4.28–9.19)		1.10 (0.63–1.93)	0.739		–	–
Overseas travel									
No	416	14	3.37 (1.63–5.10)		†	†		†	†
Yes	2,821	180	6.38 (5.48–7.28)		1.96 (1.12–3.40)	0.017		1.24 (0.69–2.25)	0.471
Previous malaria deferral									
No	1,684	74	4.39 (3.42–5.37)		†	†		†	†
Yes	1,553	120	7.73 (6.40–9.06)		1.82 (1.35–2.45)	0.000		1.80 (1.31 −2.47)	0.000
Previous diarrhea deferral									
No	3,179	185	5.82 (5.01–6.63)		†	†		†	†
Yes	58	9	15.52 (6.20–24.84)		2.97 (1.44 – 6.14)	0.003		2.55 (1.22–5.33)	0.013
Donor status									
New	307	13	4.23 (1.98–6.49)		†	†		–	–
Repeat	2,930	181	6.18 (5.31–7.05)		1.49 (0.84–2.65)	0.175		–	–

*ACT, Australian Capital Territory; HEV, hepatitis E virus; NSW, New South Wales; NT, Northern Territory; OR, odds ratio; QLD, Queensland; SA, South Australia; TAS, Tasmania; VIC, Victoria; WA, Western Australia; –, indicates factor was not included in multivariate analyses. †Reference group used in respective analyses.

Detection of HEV IgM in 4 (2.06%) of the 194 samples from IgG-positive donors indicates the donors had been recently exposed to HEV (95% CI 0.06–4.06). All 4 donors had traveled overseas; 3 reported travel to malaria-endemic countries. HEV RNA was not detected in any of the HEV IgG–positive samples. Although it is encouraging that HEV nucleic acid was not detected, the sample size is insufficient to accurately determine the true rate of HEV RNA carriage among donors in this study; a larger study is planned.

Management strategies to safeguard the Australian blood supply against transfusion-transmitted HEV are based on donor selection guidelines. To identify donors with possible bacteremia/viremia, including HEV, blood donation staff members ask donors several medical, behavioral, and travel-based questions before donation. These include questions relating to general wellness, sex practices, gastric upset, diarrhea, abdominal pain, and vomiting within the previous week. In addition, for 4 months after a donor’s return from travel to a malaria-endemic country, donations are restricted to plasma for fractionation. Some protection against blood donations from HEV-infected persons may occur because HEV and malaria are co-endemic to many countries. Our findings showed a higher HEV seroprevalence among donors with prior malaria or diarrhea deferrals; thus, malaria- and diarrhea-related screening questions may reduce contributions from donors with travel-associated HEV infection.

Our findings showed HEV exposure in travelers and nontravelers, suggesting the possibility of imported and locally acquired HEV in Australia. Prior HEV exposure was higher in donors who were temporarily excluded from donating blood on previous donation attempts, suggesting the current management strategy in Australia is partially effective in minimizing any risk of HEV transmission through blood transfusion. However, the presence of HEV IgG in donors who reported no overseas travel and/or no prior related deferrals, coupled with the knowledge that asymptomatic infection is possible, suggests that additional safety precautions may be warranted.
